# Mating compatibility in the parasitic protist *Trypanosoma brucei*

**DOI:** 10.1186/1756-3305-7-78

**Published:** 2014-02-21

**Authors:** Lori Peacock, Vanessa Ferris, Mick Bailey, Wendy Gibson

**Affiliations:** 1 School of Biological Sciences University of Bristol, Bristol BS8 1UG, UK; 2 School of Clinical Veterinary Science, University of Bristol, Langford, Bristol BS40 7DU, UK

## Abstract

**Background:**

Genetic exchange has been described in several kinetoplastid parasites, but the most well-studied mating system is that of *Trypanosoma brucei*, the causative organism of African sleeping sickness. Sexual reproduction takes place in the salivary glands (SG) of the tsetse vector and involves meiosis and production of haploid gametes. Few genetic crosses have been carried out to date and consequently there is little information about the mating compatibility of different trypanosomes. In other single-celled eukaryotes, mating compatibility is typically determined by a system of two or more mating types (MT). Here we investigated the MT system in *T. brucei*.

**Methods:**

We analysed a large series of F1, F2 and back crosses by pairwise co-transmission of red and green fluorescent cloned cell lines through experimental tsetse flies. To analyse each cross, trypanosomes were cloned from fly SG containing a mixture of both parents, and genotyped by microsatellites and molecular karyotype. To investigate mating compatibility at the level of individual cells, we directly observed the behaviour of SG-derived gametes in intra- or interclonal mixtures of red and green fluorescent trypanosomes *ex vivo*.

**Results:**

Hybrid progeny were found in all F1 and F2 crosses and most of the back crosses. The success of individual crosses was highly variable as judged by the number of hybrid clones produced, suggesting a range of mating compatibilities among F1 progeny. As well as hybrids, large numbers of recombinant genotypes resulting from intraclonal mating (selfers) were found in some crosses. In *ex vivo* mixtures, red and green fluorescent trypanosome gametes were observed to pair up and interact via their flagella in both inter- and intraclonal combinations. While yellow hybrid trypanosomes were frequently observed in interclonal mixtures, such evidence of cytoplasmic exchange was rare in the intraclonal mixtures.

**Conclusions:**

The outcomes of individual crosses, particularly back crosses, were variable in numbers of both hybrid and selfer clones produced, and do not readily fit a simple two MT model. From comparison of the behaviour of trypanosome gametes in inter- and intraclonal mixtures, we infer that mating compatibility is controlled at the level of gamete fusion.

## Background

Discrimination of self and non-self by the molecular recognition of cells of the same or different genotype is a fundamental attribute of eukaryote cells. In single-celled organisms, it is of particular importance in guiding the choice of a suitable partner for mating. Mating compatibility is often controlled by a system of mating types (MT), such that mating between cells of different MT is preferred over same MT. MT systems are orchestrated by diverse molecular mechanisms and appear to have evolved independently many times [1-4]. Some systems do not exclude mating between identical genotypes, because a single lineage can produce cells of opposite MT, e.g. ciliates [5], *Plasmodium falciparum *[6]. While theoretical evolutionary studies have concluded that the optimum number of MT is two [2], more than ten are known in some ciliates [5].

Among kinetoplastid protists, genetic exchange has been described in *Trypanosoma brucei *[7], *T. cruzi *[8], *Leishmania major *[9] and *Crithidia bombi *[10]. The most well-studied mating system is that of *T. brucei*, the causative organism of African sleeping sickness, which is transmitted by an insect vector, the tsetse fly. It is during the developmental cycle of *T. brucei* within the salivary glands of the fly that mating occurs [11]. *T. brucei* is diploid during most of its life cycle [12-14] and briefly undergoes meiosis in the tsetse salivary glands, evidenced by the expression of meiosis-specific genes and production of haploid gametes [15,16]. The mitochondrial DNA is inherited biparentally [17-19]. In kinetoplastids the mitochondrial DNA has a unique and complex structure, consisting of a network of intercalated circular DNA molecules densely packed into an organelle called the kinetoplast [20]. The biparental inheritance of kinetoplast DNA (kDNA) has implications for the inheritance of cell organelles, because the kinetoplast is structurally linked to the basal body of the single flagellum via a tripartite attachment complex [21]. Thus, it follows from the observation that hybrid progeny have hybrid kinetoplast DNA networks, that zygote formation involves fusion of cell bodies and not just the exchange of nuclei as seen for example in ciliates [5].

In *T. brucei* intraclonal mating (= selfing) occurs at very low frequency compared to interclonal mating (= outcrossing) [22-24], implying that there is discrimination between self and non-self at some level. Little is known about mating compatibility, because so few trypanosome crosses have been carried out, but there appear to be at least three MT judging by the success of pairwise crosses of three genetically unrelated strains which all successfully produced hybrid progeny [25]. Nothing is yet known about the molecular basis of mating compatibility in *T. brucei* and the genes determining MT remain unidentified. Recently, the analysis of trypanosome crosses has been facilitated by the incorporation of red or green fluorescent tags into the parental trypanosome clones, enabling identification of flies containing mixed infections and hybrid progeny by dual fluorescence [11,24,26]. Here, we have exploited this red/green system to investigate mating compatibility in trypanosomes by analysing a series of F1, F2 and back crosses. To investigate mating compatibility at the level of individual cells, we directly observed the behaviour of gametes in intra- or interclonal mixtures of red and green fluorescent trypanosomes *ex vivo*.

## Methods

### Trypanosomes

The trypanosome clones used were the parents and four F1 hybrid clones from the cross described previously [11]. The parents were 1738 (*T. b. brucei* MOVS/KE/70/1738; [27]) and J10 (*T. b. brucei* MCRO/ZM/73/J10 CLONE 1; [28]), transfected with either a gene for enhanced GFP or modified RFP [29] in plasmid constructs designed to integrate into the non-transcribed spacer in the trypanosomal ribosomal RNA locus [30,31]. F1G1 and F1G2 were two diploid, green fluorescent F1 clones, while F1R1 and F1R2 were two diploid, red fluorescent F1 clones [11]. In addition, F2G1 and F2R1 were diploid green or red fluorescent F2 hybrids respectively from cross F1G2 x F1R1 (cross C, Table 1) carried out here.

**Table 1:** F1 crosses and back crosses

**Cross**	**Total no. of clones**	**DNA content**^**a**^			**Hybrids (H)**		**Selfers**^**b **^**(S)**		**Parent (P)**		**% clones**		
		**No.**	**2 N**	**>2 N**	**No.**	**Genotypes**	**No.**	**Genotypes**	**2 N**	**>2 N**	**H**	**S**	**P**
J10 RFP x 1738 GFP^c^	63	29	19	10	63	29	0	0	0	0	100%	0%	0%
**F1 crosses**													
A: F1G1 x F1R1	20	19	4	15	7	7	3	3 (F1G1)	0	5 F1R1, 5 F1G1	35%	15%	50%
B: F1G2 x F1R2	8	5	5	0	3	2	2	1 (F1R2)	3 F1G2	0	38%	25%	38%
C: F1G2 x F1R1	13	13	11	2	11	9	1	1 (F1R1)	1 F1R1	0	85%	8%	8%
D: F1G1 x F1R2	12	12	5	7	1	1	4	3 (F1G1)	3 F1G1	4 F1G1	8%	33%	58%
**Back crosses**													
E: F1G1 x J10 RFP	15	15	10	5	5	3	1	1 (F1G1)	6 F1G1	3 F1G1	33%	7%	60%
F: F1G1 x 1738 RFP	11	11	1	10	8	5	3	2 (F1G1)	0	0	73%	27%	0%
G: F1G2 x J10 RFP	24	23	11	12	12	9	1	1 (F1G2)	6 F1G2	5 F1G2	50%	4%	46%
H: F1G2 x 1738 RFP	10	9	2	7	0	0	6	5 (F1G2)	1 F1G2	3 F1G2	0%	60%	40%
I: F1R1 x J10 GFP	7	7	5	2	2	2	5	2 (F1R1)	0	0	29%	71%	0%
J: F1R1 x 1738 GFP	18	15	12	3	4	1	0	0	12 F1R1	2 F1R1	22%	0%	78%
K: F1R2 x J10 GFP	33	30	27	3	3	2	24	5 (F1R2)	4 F1R2	2 F1R2	9%	73%	18%
L: F1R2 x 1738 GFP	6	4	3	1	6	4	0	0	0	0	100%	0%	0%

^a^DNA contents were classified as 2 N (mean 1.02, range 0.78 – 1.17; N = 98) or >2 N (mean 1.68, range 1.21 – 2.14; N = 67) standardized against the DNA content (= 1.00) of a control sample *T. b. brucei* 427.

^b^Includes clones that have homozygous microsatellite alleles where the parent was heterozygous, clones that are identical to the parent except that they have lost the GFP gene, clones that are identical to the parent except that they have a non-parental karyotype. Parent of origin in brackets.

^c^Results taken from [11].

### Experimental tsetse transmission

**Figure 1:**
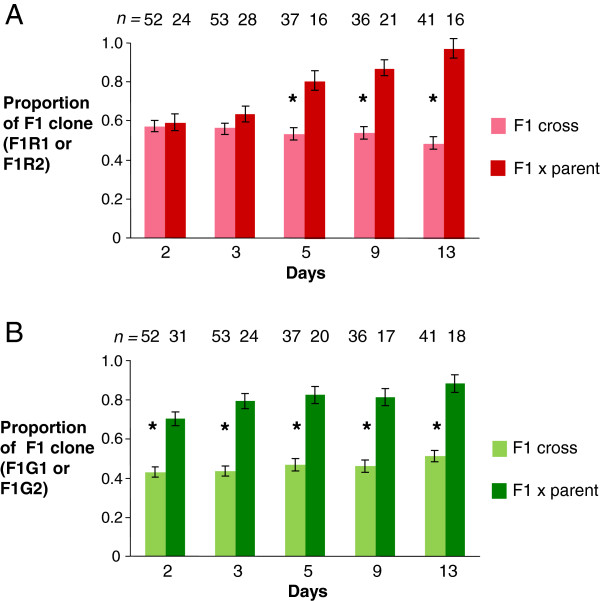
**Relative proportions of parental and F1 trypanosomes in midguts.** Flies were fed approximately equal numbers of bloodstream form trypanosomes and dissected on the days indicated. **A**. Combined results for F1R1 and F1R2 crossed with either an F1 hybrid clone (F1G1 or F1G2) or a parental clone (J10 GFP or 1738 GFP). **B**. Combined results for F1G1 and F1G2 crossed with either an F1 hybrid clone (F1R1 or F1R2) or a parental clone (J10 RFP or 1738 RFP). *n* = number of flies. For F1R1/F1R2, F = 93.842, *P* < 0.001, and for F1G1/F1G2, F = 227.208, *P* < 0.001, showing that in both cases there was a significantly higher proportion of the F1 clone in the back crosses compared to F1 crosses over time.

Experimental tsetse flies (*Glossina morsitans morsitans* or *G. pallidipes*) were reared and infected essentially as described previously [11,24]. Male flies were used for experiments, being given the infective bloodmeal for their first feed 24–48 hours post-eclosion. The infective bloodmeal contained approximately equal numbers of cryopreserved, bloodstream form trypanosomes of each clone (estimated 8 × 10^6^ trypanosomes ml^-1^) in sterile horse blood supplemented with 60 mM N-acetylglucosamine [32] or 10 mM L-glutathione [33] to increase infection rates. For the back crosses, initial trials suggested that the parental clones were outcompeted by the F1 hybrid clones, because many midgut infections showed low numbers of the parental trypanosome relative to the F1 clone (Figure 1). This imbalance was also evident in the salivary gland populations, with few, if any, mixed infections. To boost numbers of the parental clones in these crosses, we used *in vitro* cultured procyclics in washed red blood cells instead of bloodstream forms, and thus obtained higher ratios (between 10:1 to 100:1) of parental clone to F1 clone in the infective feed.

Flies were dissected in a drop of phosphate buffered saline (PBS) 33 to 59 days after infection and organs (midgut, salivary glands) were examined for the presence of trypanosomes by phase contrast microscopy. Infected organs were viewed by fluorescence microscopy to determine the colours of trypanosomes present. Salivary glands containing an approximately equal mixture of trypanosome clones as judged by fluorescence were taken forward for isolation of hybrids. For midgut counts, each infected midgut was placed in a microcentrifuge tube containing 50 μl PBS and thoroughly disrupted using a Teflon pestle. The trypanosomes were fixed by adding paraformaldehyde to a final concentration of 0.1% w/v in PBS and red and green trypanosomes counted under fluorescence using a haemocytometer to determine the relative proportions.

### Isolation and analysis of hybrid progeny

Metacyclics from infected salivary glands were inoculated into mice. Bloodstream forms from the first peak of parasitaemia were harvested and transformed into procyclics *in vitro* by incubation in Cunningham’s medium (CM) [34] at 27°C. Clones were obtained by limiting dilution of procyclics in CM in 96 well plates incubated at 27°C in 5% CO_2_. The colour of each clone was determined by fluorescence microscopy. From each clone a genomic DNA sample was prepared using a spin column (Qiagen) and a sample for pulsed field gel (PFG) electrophoresis was prepared by lysing and deproteinising trypanosomes *in situ* in agarose blocks [35]. For each cross we usually analysed 6–8 clones from each of two flies harbouring a mixed infection in the salivary glands.

Microsatellite analysis was carried out essentially as described previously [11,24] using four to eight microsatellite loci [36]. PFG electrophoresis and hybridisation was carried out as previously described [11,24]; each blot was hybridised with informative DNA probes derived from genes for β-tubulin [chromosome (chr.) I], trypanothione synthetase (TS; chr. II), PFR1 (chr. III), RFP [integrated into ribosomal RNA (rRNA) locus on chr. III], P67 (chr. V), KRET1 (chr. VII), GFP (integrated into rRNA locus on chr. VII), 5S rRNA (chr. VIII), 18S rRNA (multiple chromosomes). Kinetoplast DNA maxicircle type was determined for selected clones as previously described [11].

### Measurement of DNA contents

DNA contents were measured by flow cytometry of propidium iodide stained procyclic cells essentially as described previously [11], except that all measurements were standardized against the DNA content (= 1.0) of a control sample *T. b. brucei* 427, which was prepared and run with each batch of test samples.

### *Ex vivo* mixing experiments

Batches of tsetse were infected with *T. brucei* clones J10 RFP, 1738 GFP, 1738 RFP, F1G2 or F1R1. After 15–21 days, 10–15 flies infected with each clone were dissected and the salivary glands immediately placed in 50 μl of CM, allowing unattached trypanosomes to spill out into the medium with the saliva. The trypanosome suspension was then transferred to a clean tube. Small aliquots (10–20 μl) of different clones were mixed and introduced into a microslide capillary tube. The microslide was systematically searched along its length by phase and fluorescence microscopy. Interacting pairs or clusters of trypanosomes were recorded, noting the colours of cells in each cluster, together with the presence of any yellow fluorescent hybrid cells.

### Statistical analysis

Results of the individual midgut counts were analysed using a general linear model with clone type (F1 hybrid or parent) and time as factors. Pairwise comparisons of the proportions of the F1 hybrid clone mixed with another F1 or parent clone over time were made by post-hoc comparisons using the least significant difference.

### Ethical issues

Animal experiments were carried out under a UK Home Office Project licence in accordance with the current legislation on standards of welfare for experimental animals.

## Results

### Analysis of F1 crosses

**Figure 2:**
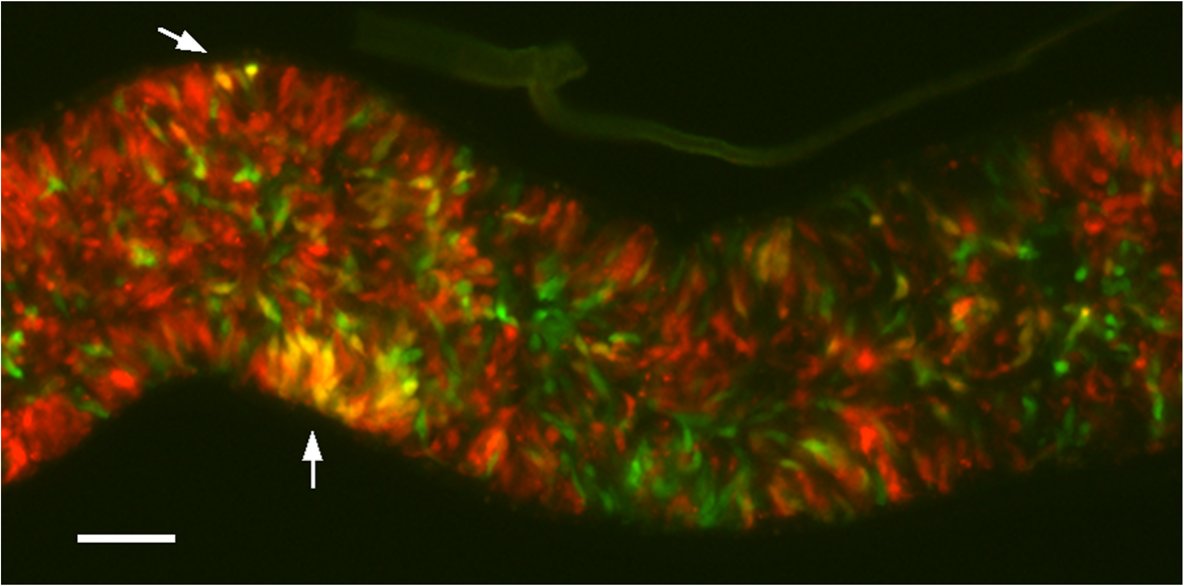
**Salivary gland containing hybrids.** Part of tsetse salivary gland packed with red (F2R1) and green (J10 GFP) fluorescent trypanosomes 34 days after infection (cross P, Table 2). Yellow fluorescent trypanosomes are present (arrows). Scale bar = 50 μm.

**Figure 3:**
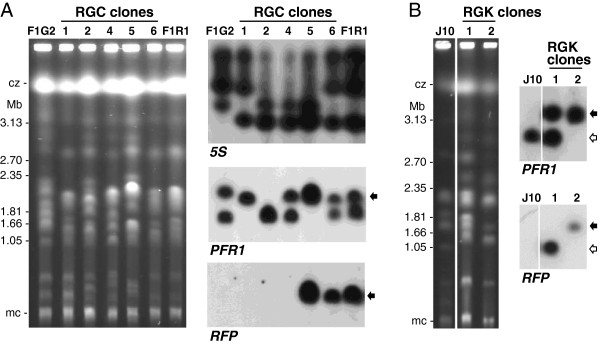
**Molecular karyotypes of progeny clones. A**. Ethidium bromide stained gel comparing the molecular karyotypes of clones from cross C (F1G2 x F1R1). Size marker: chromosomal DNA from *Hansenula wingei*; cz = compression zone, a region of the gel where several large chromosomal bands are trapped; mc = minichromosomes of approx. 100 kb in size. Clones 1, 2, 4 and 5 are hybrid, while clone 6 has a karyotype identical to that of parent F1R1. Other panels show autoradiographs following hybridization with the probes indicated. All blots were washed to 0.1 × SSC at 65°C. The arrows indicate the chromosome III homologue that carries both the *PFR1* and *RFP* genes. **B**. Ethidium bromide stained gel comparing the molecular karyotypes of clones from cross K (J10 x F1R2). Clone 1 has an identical karyotype to F1R2 (not shown), while clone 2 has a different karyotype to both F1R2 and J10. The other panels show autoradiographs following hybridization with the probes indicated. PFR1 hybridization reveals that clone 1 and F1R2 (not shown) have chromosome III homologues of different sizes, whereas in clone 2 the bands co-migrate. The arrows indicate the chromosome III homologue that carries both the *PFR1* and *RFP* genes in clone 1 (clear arrow) and clone 2 (filled arrow). Clone 2 had only genetic input from F1R2 and is a selfer.

**Figure 4:**
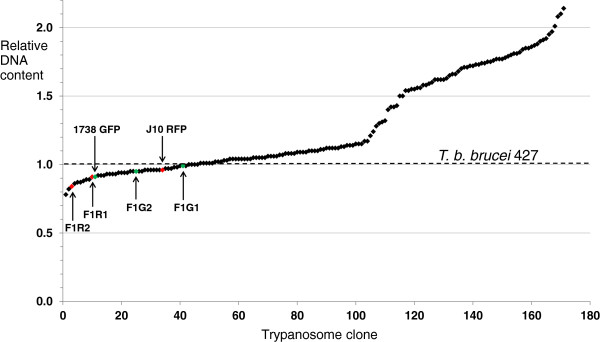
**DNA contents of parental and progeny clones.** Procyclic trypanosomes were stained with propidium iodide and the peak intensity of red fluorescence was measured by flow cytometry. All values were normalized relative to *T. b. brucei* 427, which was included in each run. DNA contents of individual clones (diamonds) are plotted on an arbitrary scale relative to the DNA content of *T. b. brucei* 427 = 1.0 (dotted line). The two parental clones, J10 and 1738, and four F1 clones are indicated; all have slightly lower DNA contents than the standard, *T. b. brucei* 427.

Two green fluorescent diploid F1 clones, F1G1 and F1G2, and two red fluorescent diploid clones, F1R1 and F1R2, derived from our previous cross of *T. b. brucei* J10 RFP and 1738 GFP [11], were crossed in all possible red x green combinations by tsetse co-transmission (crosses A to D; Table 1). For each cross, we identified two flies with infected salivary glands (SGs) containing both red and green fluorescent trypanosomes (only one fly for cross B); in most cases, yellow fluorescent trypanosomes were also observed in these infected SGs, indicating that mating had taken place between the red and green parental trypanosomes (Figure 2). Progeny from each fly SG population were cloned and genotyped by microsatellite analysis and PFG karyotype (Figure 3). Selected clones, representing all the genotypes found, were also analysed for DNA content (Figure 4). Results for these F1 crosses are summarized in Table 1, together with data from the J10 RFP x 1738 GFP cross for comparison. All four F1 crosses produced at least one hybrid progeny clone, but cross C (F1G2 x F1R1) was particularly successful as 85% of clones were hybrid (Table 1, Figure 3A).

In addition to hybrid clones, in each cross there was a proportion of progeny (8-33% of clones) that appeared to be the product of intraclonal mating (selfers), in that genetic reassortment indicative of genetic exchange was evident from PFG karyotype and/or microsatellite alleles, but genetic input from only one parent was demonstrable (e.g. Figure 3B). Kinetoplast DNA maxicircle type was also analysed in cases where this differed between parental clones, because some hybrid progeny have been found previously where only kDNA had been exchanged [37]; however, no such instance was found here and all selfer clones had kDNA maxicircles derived from their parental clone of origin.

Clones that were genetically indistinguishable from the parents were also recovered from each cross (Table 1). Some of these had elevated DNA contents, suggesting that they might be the result of intraclonal mating, but supporting evidence of homozygosity at any of the microsatellite loci examined was lacking. The numbers of each type of progeny clone recovered from individual crosses are summarized in Table 1.

**Figure 5:**
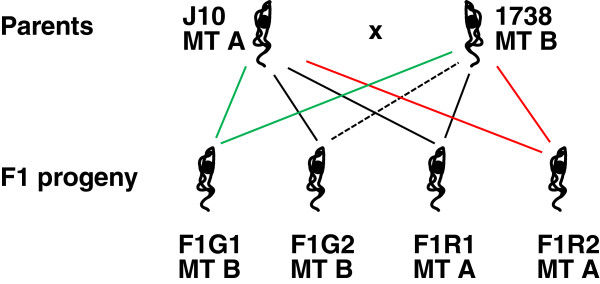
**A two mating type (MT) system does not fit the data.** In the diagram, parental trypanosomes J10 and 1738 are arbitrarily designated MT A or B. The four F1 progeny clones successfully produced hybrids when crossed in all pairwise red/green combinations and therefore, in a two MT system, the green and red F1 clones should be of different MT as shown. The model predicts that half the back crosses should fail, because only clones of different MT are compatible; however, seven of the eight back crosses successfully produced hybrids (Table 1). Therefore a two MT system does not fit the data. Solid and broken lines indicate back crosses that produced or did not produce hybrid progeny respectively.

Considering a two MT model, in which parental strains J10 and 1738 are assumed to be of different MT, A and B, then the success of each of the four F1 crosses implies that the green F1 clones are of different MT to the red F1 clones, as shown in Figure 5. Given that each clone has committed to the expression of a single MT, A or B, then half the back crosses of F1 progeny to parent would be unsuccessful (dotted lines), because the MT will be identical. We set up a series of back crosses to test this.

### Analysis of back crosses

Each of the four F1 progeny clones was crossed with each parental strain, J10 and 1738, in red x green combinations (crosses E to L, Table 1). For each back cross we analysed two flies with infected SGs containing both red and green fluorescent trypanosomes (only one fly for crosses I and L, three for cross J); in some cases, yellow fluorescent trypanosomes were also observed in one or both of these infected SGs (crosses E, F, J and K; Figure 2). Progeny from each fly SG population were cloned and genotyped, and DNA contents of representative clones were measured. Results for the eight back crosses are summarized in Table 1. All back crosses except one (H: F1G2 x 1738 RFP) successfully produced hybrids; yellow fluorescent trypanosomes were not observed in the SG for cross H and all the progeny clones isolated were green fluorescent. Under the scheme in Figure 5, this cross involved two clones of the same MT (MT B), and was thus predicted to fail because of self-incompatibility, along with three other same MT crosses (F, I and K). However, these three crosses all produced hybrids (Table 1).

As a final test of this simple, two fixed MT model, we crossed two F2 progeny clones from F1 cross C (F1G2 x F1R1) together and in all possible red x green combinations with the two parental strains, J10 and 1738, using both red and green fluorescent clones of each (crosses M to Q, Table 2). Progeny were cloned and genotyped as before and results are summarized in Table 2. The cross of the F2 progeny (cross M) was successful, indicating that clones F2G1 and F2R1 are of different MTs. All the back crosses to parents J10 and 1738 also successfully produced hybrid genotypes (Table 2). But this does not fit the model, which predicts that only half of these crosses will be compatible.

**Table 2:** F2 crosses and back crosses

**Cross**	**Total no. of clones**	**Hybrids (H)**		**Selfers (S)**		**Parental (P)**		**% Clones**		
		**No.**	**Genotypes**	**No.**	**Genotypes**	**No.**	**Genotypes**	**H**	**S**	**P**
M: F2G1 x F2R1	10	7	5	0	0	3	2	70%	0%	30%
N: F2G1 x J10 RFP	5	5	3	0	0	0		100%	0%	0%
O: F2G1 x 1738 RFP	8	5	3	0	0	3	All F2G1	63%	0%	38%
P: F2R1 x J10 GFP	10	10	6	0	0	0		100%	0%	0%
Q: F2R1 x 1738 GFP	15	12	8	3	1 (1738)	0		80%	20%	0%

In summary, our results are not consistent with this model, where the two MT’s are fixed in each trypanosome clone and the outcome of crosses is binary – success or failure (Figure 5). The outcomes of individual crosses, particularly back crosses, were much more variable than this model predicts. Not only were numbers of hybrid clones very variable, but there were also large differences in the numbers of selfer and parental clones (Table 1). The number of selfer clones varied inversely with the number of hybrid clones, suggesting that selfing occurs more frequently in less compatible crosses. We next investigated how this pattern might have arisen by direct observation of gamete interactions.

### Direct observation of gamete interactions

*T. brucei* undergoes meiosis and produces haploid gametes as part of the normal development of a single trypanosome clone in the tsetse salivary glands [15,16]. The gametes are morphologically distinctive, with a compact, pear-shaped body and a long flagellum. In mixtures of compatible trypanosomes such as J10 RFP/1738 GFP or F1G2/F1R1, gametes interact by intertwining their long flagella and after cytoplasmic exchange, produce yellow fluorescent cells [16].

**Figure 6:**
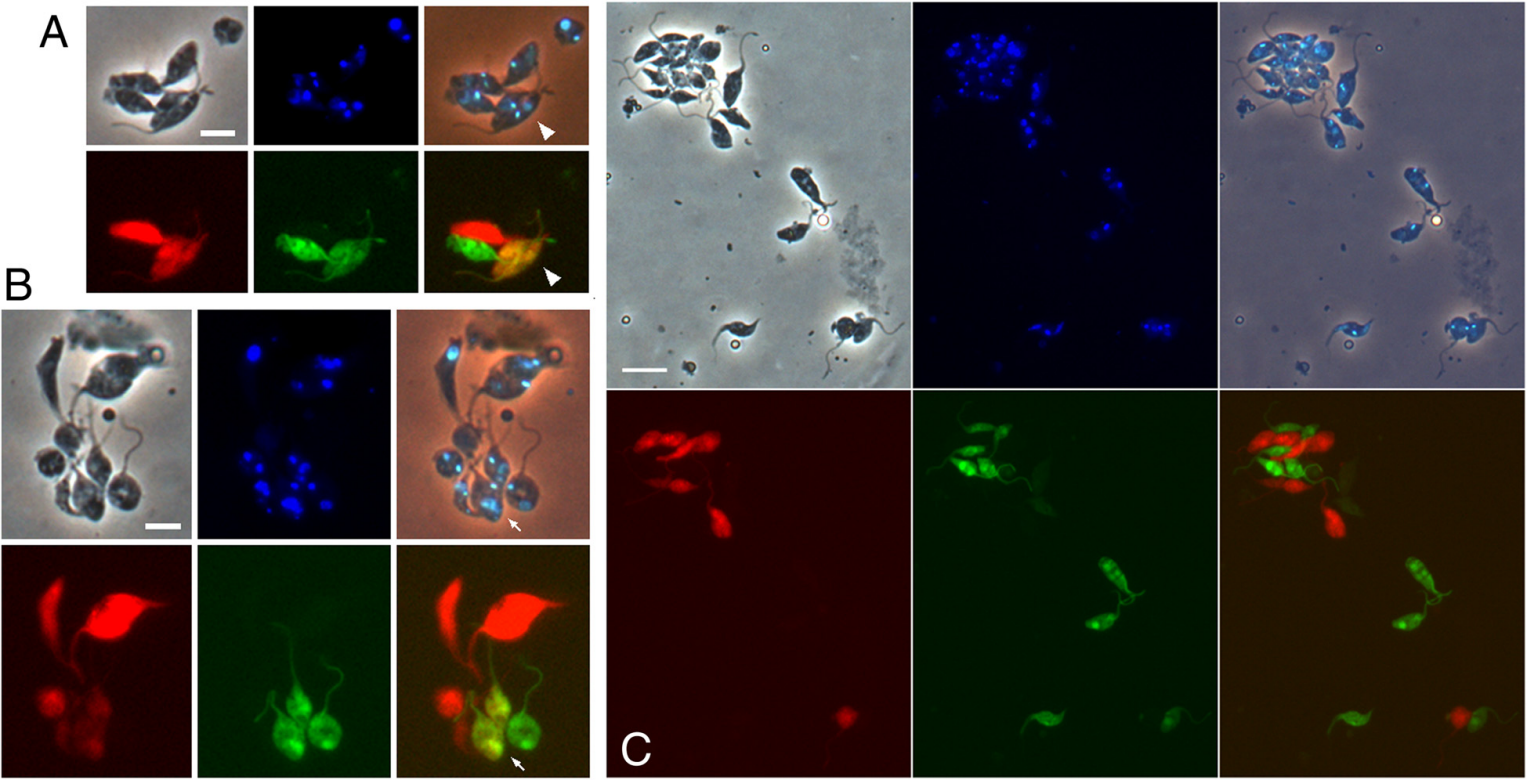
**Interactions and cytoplasmic exchange between trypanosome gametes.** Red and green fluorescent trypanosomes derived from tsetse salivary glands were mixed *ex vivo* and fixed after a period of approximately 30 minutes. **A**. Cluster of interacting trypanosomes in a mixture of F1G2 (green fluorescence) and 1738 RFP (red fluorescence). Two yellow fluorescent trypanosomes are arrowed. Scale bar 5 μm. **B**. Cluster of interacting trypanosomes in a mixture of F1G2 (green fluorescence) and F1R1 (red fluorescence). Two adjacent yellow fluorescent trypanosomes are indicated by the arrow. These two yellow trypanosomes and the adjacent green trypanosome display the typical morphology of trypanosome gametes with a short, pear-shaped body and long flagellum. Scale bar 5 μm. **C**. Large cluster and two pairs of interacting trypanosomes from the intraclonal mixture of 1738 RFP and 1738 GFP. Many of these trypanosomes show the typical gamete morphology (pear-shaped body, long flagellum), particularly the red-green pair bottom right. No yellow trypanosomes are present. Scale bar 10 μm.

Here, we investigated whether these characteristic gamete interactions also occur in intraclonal mixtures of 1738 RFP/1738 GFP (Table 3). A total of 15 replicates were carried out comparing the interactions of 1738 GFP with either 1738 RFP or J10 RFP in the same experiment. In both interclonal (1738 GFP x J10 RFP) and intraclonal (1738 GFP x 1738 RFP) mixtures, red and green fluorescent trypanosomes were observed to form clusters of two or more cells from approximately 10 minutes after mixing (Figure 6). Cell-cell interactions involved the intertwining of the long flagella of the gametes and close proximity of the cell bodies (Additional file 1). No differences were apparent between trypanosome clusters in interclonal mixtures (Figure 6A and B) compared to intraclonal mixtures (Figure 6C), except in the frequency of yellow fluorescent trypanosomes. These result from cytoplasmic mixing of red and green fluorescent proteins and hence indicate that fusion between red and green fluorescent trypanosomes has occurred [16]. Yellow fluorescent trypanosomes were frequently encountered in interclonal mixtures in which interacting red and green fluorescent trypanosomes (RG clusters) were present (Figure 6A and B; Table 3) and were typically observed within 10–30 minutes of mixing the red and green fluorescent trypanosomes. In contrast, for the intraclonal mixture (1738 RFP/1738 GFP), yellow fluorescent trypanosomes were observed in only one of 9 experiments with RG clusters (Table 3), and this was towards the end of the experiment after observation of the *ex vivo* mixture for well over an hour. Yellow trypanosomes were also observed in all 5 experiments with F1G2/F1R1 (cross C; results reported previously in [16]) and one of 5 experiments with F1G2/1738 RFP (cross H) (Table 3). Although numbers are small, the relative frequency of yellow trypanosomes in these *ex vivo* mixtures reflects the relative numbers of hybrid clones recovered from the same *in vivo* crosses (Table 1).

**Table 3:** Trypanosome interactions in pairwise mixing of salivary gland (SG) derived trypanosomes

**Mixture**		**Number of experiments**		
**Red**	**Green**	**Total**	**No. with Red-Green clusters**	**No. with yellow cells**
1738 RFP	1738 GFP	15	9	1^a^
J10 RFP	1738 GFP	15^b^	11	6
F1R1	F1G2	5^b^	5	5
1738 RFP	F1G2	5	5	1

^a^Observed at end of experiment after > 1 hour of observation.

^b^Results taken from [16].

In summary, while gamete behaviour and the number of RG clusters was similar in intra- and interclonal mixtures, yellow fluorescent trypanosomes were very rare in intraclonal compared to interclonal mixtures, indicating that gamete interactions and gamete fusion are two separate processes.

## Discussion

While hybrids are readily found in mixtures of compatible *T. brucei* strains [25,38,39], intraclonal mating is rare [22-24]. Therefore *T. brucei* can discriminate between self and non-self and hence has a system of mating types (MTs). The simplest system is two MT [1], where MT A must cross with MT B (Figure 5). The data presented here do not fit a model where each trypanosome clone is committed to always express a single MT. Instead, the success of individual crosses was highly variable in terms of the relative number of hybrid and selfer clones produced (Tables 1 and 2), and outcome could not be easily categorized in a binary scheme of success or failure. The wide range of mating success observed in different crosses indicates that the F1 clones have different mating compatibilities to the parents. For example, F1 cross C (F1G2 x F1R1) was almost as successful as the parental cross (J10 RFP x 1738 GFP) in the percentage of hybrid clones produced, while crosses A (F1G1 x F1R1) and B (F1G2 x F1R2) were of moderate success and cross D (F1G1 x F1R2) produced only one hybrid clone (Table 1). Crosses that produced abundant hybrid clones tended to produce low numbers of intraclonal recombinants (selfers), and *vice versa* (e.g. crosses C, F and L versus crosses D, H, I and K; Table 1).

The differences observed here in the relative numbers of hybrid clones recovered from individual crosses are likely to reflect true variation in mating compatibility rather than random chance, because of the way the trypanosome crosses were set up. The probability of isolating hybrids was maximised, because we analysed only SG that contained approximately equal numbers of both red and green fluorescent parental trypanosomes, a prerequisite for production of hybrids. For example, in our previous cross of J10 RFP and 1738 GFP [11], 22 of 60 flies with infected SG had one or both glands with a mixed infection, and yellow (hybrid) trypanosomes were seen in 17 of these 22 flies; the 5 flies without yellow trypanosomes had either been dissected too early (14 – 17 days), or had SG containing very low numbers of one of the parents. That said, it is not feasible to recover all hybrid progeny from a trypanosome cross, as for example with yeast, so the progeny clones that are eventually analysed represent only a fraction of the trypanosomes present in the SG.

We can consider our data in the light of what is known about the mating systems of other single-celled eukaryotes, with the caveat that trypanosomes are evolutionarily distant since they belong to the early diverging supergroup Excavata. Mechanistically the minimum requirement for mating between two cells is reciprocal recognition of a molecule produced by one partner and a corresponding receptor on the other partner. The interaction of these two molecules triggers a cascade leading to cell fusion, zygote formation and onward development. This is exemplified by the mating system of the flagellate *Chlamydomonas reinhardtii* where fusion occurs among plus and minus haploid gametes; the diploid zygote is heterozygous at the MT locus, so that meiosis leads to equal numbers of plus and minus gametes; tight linkage between the genes specific for each MT ensures that they are inherited together [40]. Unlike *Chlamydomonas*, *T. brucei* is diploid throughout its life cycle with a transient haploid phase [12,16]. If trypanosomes had a two determinant MT system, like *Chlamydomonas *[40] or the yeast, *Saccharomyces cerevisiae *[41], then a heterozygous diploid would produce haploid cells of either of two MT. These would be self-compatible, unless there was an additional mechanism suppressing fusion of gametes of the same genotype. Since we already know that intraclonal mating is rare for both the original parents J10 and 1738 [24], it is reasonable to assume that they are homozygous at this putative MT-determining locus, and will therefore produce heterozygous F1 progeny. Without invoking a mechanism to suppress self-compatibility, F1 and back crosses should produce hybrid and selfer progeny of each parental genotype in equal numbers. Although both hybrid and selfer clones were recovered from most of the F1 and back crosses, our data do not support this model, because selfers were typically derived from only one of the parental genotypes (Table 1).

A different MT model is seen in ciliates. Although *Tetrahymena thermophila* has seven MT, each mature ciliate commits to expression of only one set of MT-determining genes in its somatic macronucleus by deleting the other alleles present in the germline (micronucleus) [42]. *T. brucei* uses a similar strategy to select only a single variant surface glycoprotein (VSG) gene for expression in the mammalian bloodstream stage, although in this case, the arrays of silent VSG genes are not destroyed in the process. If *T. brucei* also had a repertoire of MT alleles, it could, for example, commit to expression of a single gene during the developmental programme in the vector, leading to variation in the MT expressed by a single clonal lineage. This could explain the mating compatibility patterns seen here, if different MT alleles confer variability in mating success. Interpretation will remain speculative until the MT determinants in trypanosomes have been identified and characterised at the molecular level.

Our observations of fly-derived trypanosomes *ex vivo* show that trypanosome gametes start to interact soon after they are mixed whether they are of the same or different genotype. However, cell fusion, indicated by cytoplasmic mixing, was rare unless the gametes were of different genotypes, suggesting that mating compatibility is controlled by the haploid gametes. A confounding variable in our analysis stems from the dynamics of gamete production. From our experience, different trypanosome strains vary in the abundance of gametes produced over time, and hence large numbers of one parental gamete could be present in the SG initially, in the absence of gametes from the other parent. It is also clear that there is intraspecific competition between trypanosomes within the fly that affects their development, as demonstrated here by the fact that F1 progeny clones out-competed their parents in colonising the fly midgut. Future progress will depend on obtaining sufficient gamete stages from infected flies or culture to manipulate *in vitro*.

## Conclusions

The outcomes of individual crosses, particularly back crosses, were variable in numbers of both hybrid and selfer clones produced, and do not readily fit a simple two MT model. The wide range of mating success observed here in different crosses indicates that the F1 clones have different mating compatibilities to the parents. We found no difference in the pairing of gametes and their interactions when trypanosomes were mixed intra- or interclonally. However, while cytoplasmic exchange was frequently observed in interclonal mixtures, it was rare in intraclonal mixtures. We infer that mating compatibility is controlled at the level of gamete fusion rather than gamete recognition.

## Competing interests

The authors declare that they have no competing interests.

## Authors’ contributions

WG, LP and MB designed the study. LP, VF and WG carried out the experimental work; LP and MB carried out the statistical analyses; WG and LP drafted the manuscript. All authors read and approved the final manuscript.

## Supplementary Material

Additional file 1 **Movie 1.** Intraclonal gamete interactions. 1738 RFP and 1738 GFP intraclonal mixing experiment. The two trypanosomes are interacting by intertwining their long flagella, and the cell bodies are also in close contact. Similar interactions were observed between gametes in interclonal mixtures. Phase-contrast sequence is followed by visualization of green and red fluorescence separately, followed by brief visualization of nuclei and kinetoplasts by Hoechst fluorescence. The two trypanosomes remain as separate cells and do not exchange cytoplasm. Click here for file
